# Seasonal temperatures in West Antarctica during the Holocene

**DOI:** 10.1038/s41586-022-05411-8

**Published:** 2023-01-11

**Authors:** Tyler R. Jones, Kurt M. Cuffey, William H. G. Roberts, Bradley R. Markle, Eric J. Steig, C. Max Stevens, Paul J. Valdes, T. J. Fudge, Michael Sigl, Abigail G. Hughes, Valerie Morris, Bruce H. Vaughn, Joshua Garland, Bo M. Vinther, Kevin S. Rozmiarek, Chloe A. Brashear, James W. C. White

**Affiliations:** 1 Institute of Arctic and Alpine Research, University of Colorado, Boulder, CO USA; 2 Department of Geography, University of California, Berkeley, CA USA; 3 Geography and Environmental Sciences, Northumbria University, Newcastle-upon-Tyne, UK; 4 Department of Geological Sciences, University of Colorado, Boulder, CO USA; 5 Department of Earth and Space Sciences, University of Washington, Seattle, WA USA; 6 Cryospheric Science Laboratory, NASA Goddard Space Flight Center, Greenbelt, MD USA; 7 Earth System Science Interdisciplinary Center, University of Maryland, College Park, MD USA; 8 School of Geographical Sciences, University of Bristol, Bristol, UK; 9 Climate and Environmental Physics, Physics Institute & Oeschger Centre for Climate Change Research, University of Bern, Bern, Switzerland; 10 Center on Narrative, Disinformation and Strategic Influence, Arizona State University, Tempe, AZ USA; 11 Centre for Ice and Climate, Niels Bohr Institute, University of Copenhagen, Copenhagen, Denmark; 12 College of Arts and Sciences, University of North Carolina, Chapel Hill, NC USA

**Keywords:** Palaeoclimate, Cryospheric science

## Abstract

The recovery of long-term climate proxy records with seasonal resolution is rare because of natural smoothing processes, discontinuities and limitations in measurement resolution. Yet insolation forcing, a primary driver of multimillennial-scale climate change, acts through seasonal variations with direct impacts on seasonal climate^1^. Whether the sensitivity of seasonal climate to insolation matches theoretical predictions has not been assessed over long timescales. Here, we analyse a continuous record of water-isotope ratios from the West Antarctic Ice Sheet Divide ice core to reveal summer and winter temperature changes through the last 11,000 years. Summer temperatures in West Antarctica increased through the early-to-mid-Holocene, reached a peak 4,100 years ago and then decreased to the present. Climate model simulations show that these variations primarily reflect changes in maximum summer insolation, confirming the general connection between seasonal insolation and warming and demonstrating the importance of insolation intensity rather than seasonally integrated insolation or season duration^2,3^. Winter temperatures varied less overall, consistent with predictions from insolation forcing, but also fluctuated in the early Holocene, probably owing to changes in meridional heat transport. The magnitudes of summer and winter temperature changes constrain the lowering of the West Antarctic Ice Sheet surface since the early Holocene to less than 162 m and probably less than 58 m, consistent with geological constraints elsewhere in West Antarctica^4–7^.

## Abstract (web summary)

Analysis of a continuous record of water-isotope ratios from the West Antarctic Ice Sheet Divide ice core reveals a dominant role for annual maximum insolation in determining West Antarctic summer temperature during the Holocene.

## Main

Milankovitch famously postulated that variations of Earth’s orbit and axis drive climate changes over tens of thousands of years by altering the seasonal cycle of insolation^1^. By controlling summer temperatures and ice ablation, summer insolation in the northern high latitudes is thought to drive global ice volume changes over glacial–interglacial timescales^8^. Although modelling studies support this idea^9,10^, empirical evidence of the specific climate response to insolation changes derives almost entirely from mean annual temperature reconstructions^11,12^ or from indirect effects on, for example, trapped gases and melt layers in polar ice^13,14^ and marine aeolian deposits^15^. The absence of seasonal temperature reconstructions has precluded direct evidence of insolation forcing on seasonal climate, a relationship that may vary geographically. In Antarctica, long records of multiple glacial–interglacial cycles have supported different claims about whether the effects of summer insolation relate most strongly to its maximum intensity, its seasonal integral or to duration above a threshold^2,3,16,17^. Site-specific empirical determinations would provide valuable tests of such competing ideas.

## Seasonal temperature reconstructions

**Fig. 1:**
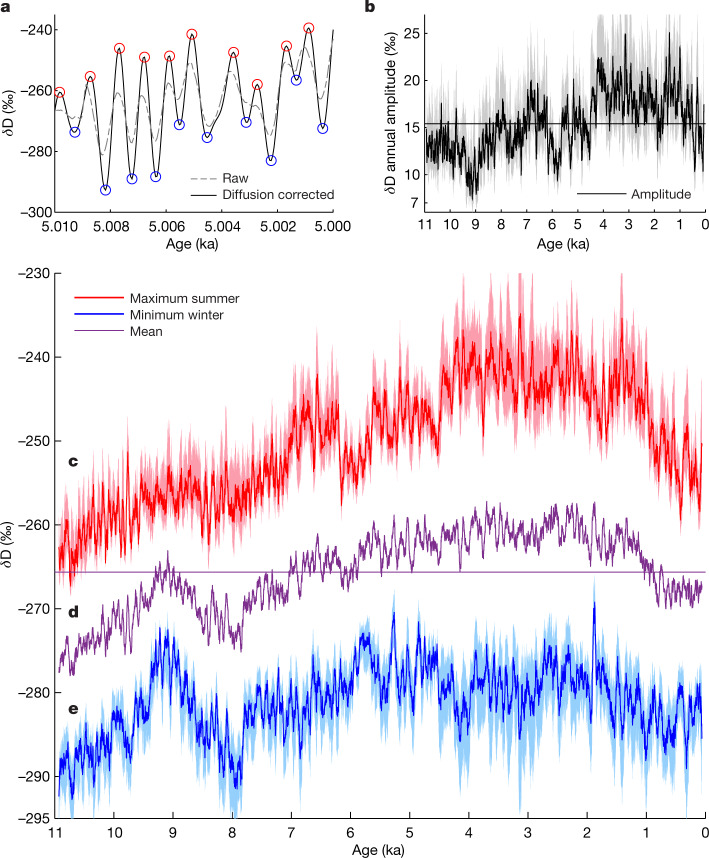
Water-isotope seasonal variability. **a**, Example section of the diffusion-corrected (solid line) and raw^20^ (dashed line) WDC δD records, with annual maxima (red circles) and minima (blue circles) determined algorithmically (Methods—Seasonal water-isotope amplitudes). Extended Data Fig. 1 provides the full high-resolution WDC δD record, diffusion lengths and extrema. **b**, The 50-yr annual-amplitude averages (summer minus winter divided by 2), with 2*σ* uncertainty; horizontal line indicates Holocene mean. **c**–**e**, The 50-yr δD averages for summer (red, **c**), mean (purple, **d**) and winter (blue, **e**); horizontal line indicates Holocene mean; shaded regions are 2*σ* bounds for combined analytical and diffusion-correction uncertainty. Source data

**Fig. 2:**
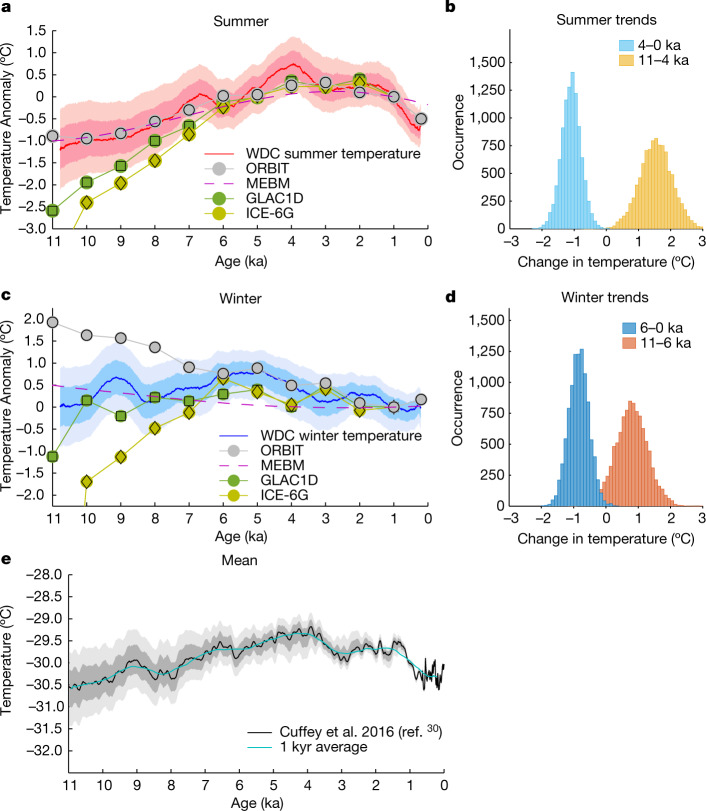
Seasonal temperature reconstruction. **a**,**c**, Reconstructed summer and winter temperatures at WDC for 1,000-yr averaging (solid red and blue lines). Shaded regions are 1*σ* and 2*σ* uncertainty ranges for combined uncertainties arising from analysis, diffusion correction, seasonality of accumulation, precipitation intermittency, isotope-temperature scaling and reconstructed mean temperatures (Methods—Uncertainties in reconstructing temperatures). Also shown are MEBM-calculated temperatures for 80° S (maximum and minimum annual values) and HadCM3 zonal temperatures for 80° S (late-December for summer, mid-August for winter) (ORBIT, GLAC1D and ICE-6G). The 0 ka ORBIT simulation uses pre-industrial settings, a calculation not available for GLAC1D or ICE-6G. Normalization is done at 1 ka when all model runs intersect within 0.05 °C and the ice-sheet configuration is well known. The ICE-6G values at 11 ka for summer and winter (not shown on plots) are −3.93 °C and −10.82 °C, respectively. Coefficient of determinations for model results versus WDC temperatures (Extended Data Fig. 7) are high for summer (HadCM3 ORBIT *R*^2^ = 0.93, *P* ≪ 0.001; MEBM *R*^2^ = 0.80, *P* ≪ 0.001) but not for winter (HadCM3 ORBIT *R*^2^ = 0.00, *P* = 0.85; MEBM *R*^2^ = 0.05, *P* = 0.30). The winter agreement improves if only the period 0–6 ka is considered (HadCM3 ORBIT *R*^2^ = 0.74, *P* = 0.01; MEBM *R*^2^ = 0.39, *P* = 0.02). **b**,**d**, Histograms of net temperature changes over the specified time intervals, derived by Monte Carlo analysis accounting for systematic and non-systematic uncertainties (Methods—Trend analysis). **e**, WDC mean annual temperature with 1*σ* and 2*σ* uncertainty bounds^30^. Extended Data Table 2 shows the amount of variability in the mean annual temperature that can be explained by the summer and winter temperatures. Source data

**Extended Data Fig. 1:**
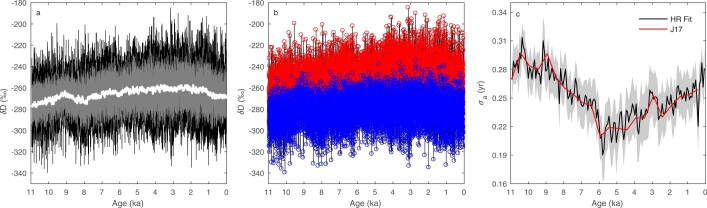
WDC water-isotope data. **a**, The raw, high-resolution WDC δD water isotope record^18–20^ (grey), the raw 50-yr running mean (white), and the diffusion-corrected signal (black). **b**, The WDC diffusion-corrected δD record with extrema picks for summer (red) and winter (blue). **c**, The high-resolution diffusion length record (black; 140-yr windows, 70-yr time steps; 1*σ* uncertainty bounds in light grey) compared to prior estimates^26^ (red; 500-yr windows, 500-yr time steps). Source data

We reconstructed seasonal temperature variability in West Antarctica through the Holocene (the last 11,000 years) and performed model experiments to understand its physical controls. The Holocene offers a window of time for assessing the influence of orbital forcing without the complicating effects of Northern Hemisphere deglaciation^18^. Our reconstruction (Figs. 1 and 2) uses the high-resolution water-isotope record (δD) from the West Antarctic Ice Sheet (WAIS) Divide ice core (WDC)^18–20^ (Methods—Water isotopes; Extended Data Fig. 1a,b), obtained with a continuous-flow technique that provides millimetre-scale depth resolution^21^. Layer ages were determined previously^2,22^.

Records of seasonal temperatures from ice cores are limited by measurement resolution and information loss from water-isotope diffusion. In Greenland, the longest records separating summer and winter variability extend to only 2 thousand years ago (ka) (refs. ^23,24^),whereas only climate model simulations are available for older periods^10^. For Antarctica, before the present study, the longest records spanned only a few centuries^25^. A combination of three factors accounts for the considerably greater scope of our reconstruction: exceptional depth resolution of measurements, conditions at WAIS Divide (high accumulation, low temperature and thick ice) which allow for preservation of subannual information through the entire Holocene^26^ and an analysis strategy which circumvents interannual noise by evaluating millennial averages of the seasonal parameters.

Our method corrects water-isotope variations for diffusion^26–28^ and assesses uncertainties including preservation bias and precipitation intermittency (Methods—Diffusion corrections and Uncertainties in reconstructing temperatures). The diffusion correction operates on the high-resolution data and produces isotopic time series from which seasonal summer–winter amplitudes were extracted. These were converted to temperature using a model-derived scaling^29^ (6.96‰ δD °C^−1^; Methods—Seasonal temperatures) and added to previously reconstructed annual mean temperatures^30^ to obtain summer and winter histories.

## Seasonal trends

**Fig. 3:**
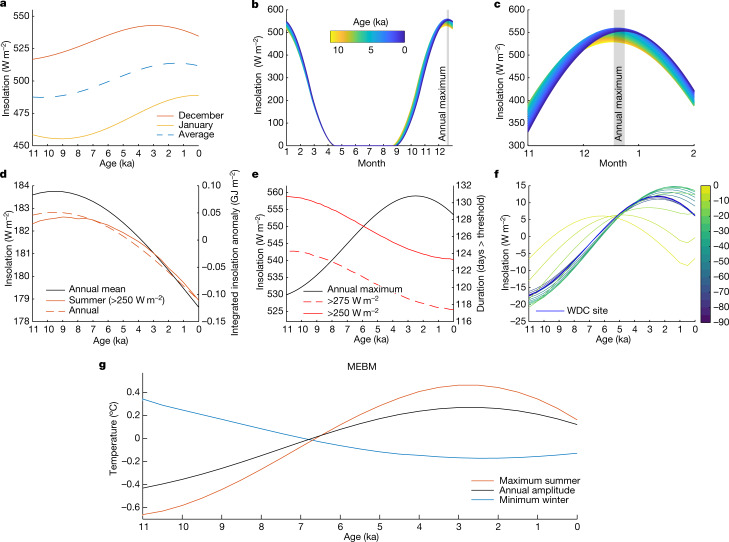
Temporal and spatial variability in insolation and model temperatures. **a**, Insolation change through the Holocene^42^ for December and January and their average. December best resembles the WDC summer reconstruction. **b**, The full seasonal cycle of insolation at 80° S for 500-yr snapshots over the Holocene. Line colours in **b** and **c** correspond to age. **c**, Zoom of summer insolation. The maximum always occurs in the latter half of December (grey shading), migrating across 8 days over the course of the Holocene. **d**, Holocene trends of annual mean insolation (black), annual integrated insolation (dashed red line) and summer-integrated insolation (red line). **e**, Maximum summer insolation intensity (black line) and summer duration (red lines), defined as the number of days above a threshold insolation value each year. **f**, Anomaly in maximum insolation coloured by latitude in the Southern Hemisphere. The thick blue line shows the latitude of the WDC site. **g**, Calculated temperatures for 80° S using the MEBM, including maximum summer value (red), minimum winter value (blue) and amplitude of the seasonal temperature cycle (black). Source data

Summer temperatures at WAIS Divide (Fig. 2a) generally rose through the early and middle Holocene, persisted at a maximum between about 5 and 1.5 ka, then decreased toward the present, with a total Holocene range of around 2 °C. These variations broadly correlate with local maximum insolation, rather than with integrated summer insolation or the duration of summer (Fig. 3d,e). Winter temperatures (Fig. 2c) varied less than summer ones overall (about 1 °C range) but also fluctuated at about 10 to 8 ka, a variation too rapid to attribute to orbital forcing.

Annual mean WAIS Divide temperature changes^30^ (Fig. 2e) were considerably influenced by winter variability in the early Holocene, whereas summer variability dominates the overall Holocene pattern (Methods—Relationship between the annual mean and individual seasons; Extended Data Table 2). Summer variability also accounts for most of the cooling in the last 2 kyr, indicating that the approximately 1 °C annual-average cooling of the entire West Antarctic during this period^31,32^ likewise reflects this season. Neither season at WDC experienced the early Holocene optimum nor overall Holocene cooling that appears in some global temperature reconstructions^33,34^. To assess the significance of the dominant multimillennial trends in each season, we performed Monte Carlo analysis (Methods—Trend analysis) using 4 ka as a demarcation point in summer (this is the timing of maximum summer temperature) and 6 ka in winter (when winter temperatures plateau). For summer (Fig. 2b) this indicates a >95% chance that warming from 11 to 4 ka and cooling from 4 ka to present exceeded 0.7 and 0.6 °C, respectively. For winter, the trend from 11 to 6 ka is indistinguishable from zero, whereas cooling of greater than about 0.3 °C from 6 to 0 ka occurred with >95% likelihood (Fig. 2d).

## Moist energy balance model

To evaluate how orbitally driven insolation changes may explain the WAIS Divide reconstructed temperatures (Fig. 2), we first simulated temperature history at 80° S using a global, zonal mean (2° resolution) moist energy balance model (MEBM) accounting for incoming and outgoing radiation, albedo and meridional atmospheric-heat transport (Methods—Moist energy balance model). The model is driven by top-of-atmosphere (TOA) seasonal insolation changes (Fig. 3a–e); for this latitude, the maximum summer insolation increases until about 2.5 ka and annual mean and annual- and summer-integrated values mostly decline through the Holocene. The calculations yield summer maximum temperatures and seasonal temperature amplitudes (Fig. 3g) that covary with local maximum summer insolation (Fig. 3e) and with the general pattern of our reconstructed summer temperatures (Extended Data Fig. 7). Although heating at lower latitudes can influence Antarctic temperature through atmospheric and oceanic heat transport, modelled maximum summer temperatures at WAIS Divide correlate best with local insolation (70° to 90° S, *R*^2^ = 0.9, *P* ≪ 0.001 during 0 to 6 ka) rather than insolation anywhere in the subtropical through subpolar latitudes (20° to 60° S, *R*^2^ = 0.33–0.55, *P* < 0.05). Indeed, models indicate heat export from WAIS Divide in summer (Extended Data Fig. 4k), rather than import from more northern locations. Since December is always the month of maximum insolation (Fig. 3a–c), variability of December insolation dominates the response of maximum summer temperature. For winter, modelled temperatures are less variable than those of summer at 80° S (Fig. 3g) because of the lack of direct insolation (Fig. 3b) and have an opposite trend. Winter minima are a function of three factors: changes in the length of the zero-insolation season, the effective cooling rate of the surface and convergent heat transport from lower latitudes. Lower minimum winter temperatures occur at times when the zero-insolation season is longer. However, neither the length of the zero-insolation season, modelled minimum temperatures, nor winter heat divergence correlate well with reconstructed winter temperatures.

## HadCM3 simulations

To investigate the role of more-complex geography and mechanisms, including topographical changes not accounted for in the MEBM, we simulated Holocene climate with a fully coupled general circulation model, HadCM3 (ref. ^35^) (Methods—HadCM3 model simulations). Simulations forced solely by changes in orbital parameters produce summer maximum temperatures (for approximately the December solstice) at 80° S similar to our reconstructed values and to the MEBM: increasing over the Holocene, peaking at 4 to 3 ka and decreasing into the modern era (ORBIT, Fig. 2a). This pattern reflects a strong role of maximum summer insolation in determining observed summer temperatures. The similarity of the early- to mid-Holocene (11–6 ka) summer temperature increase in the orbitally forced HadCM3 simulations and our reconstruction suggests little influence of changing ice-sheet elevation and extent. A similar comparison for winter yields an approximately 1.25 °C decrease of model ORBIT temperature (Fig. 2c) compared to a possible small increase in temperature in the reconstruction (Fig. 2d; >90% chance of >0.1 °C), suggesting some warming resulting from a lowering ice sheet.

**Fig. 4:**
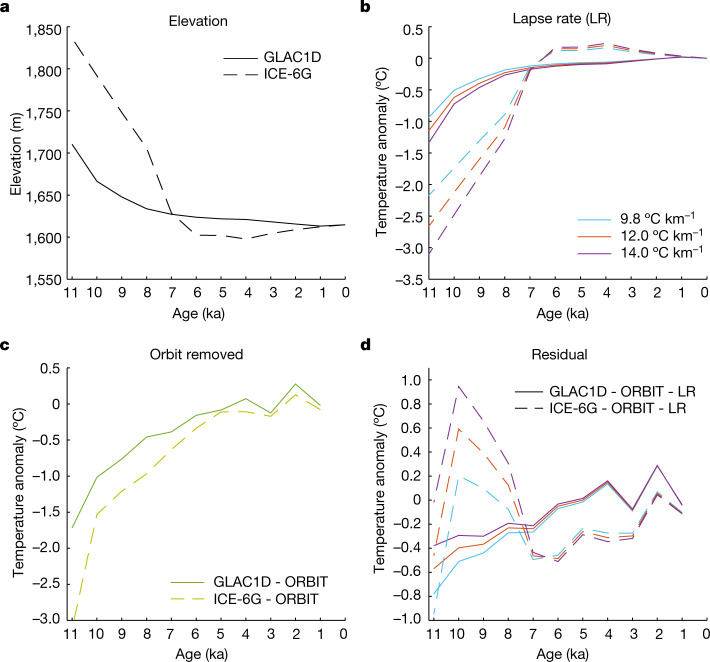
Possible ice elevation histories and the corresponding modelled temperatures. **a**, Elevation histories used in HadCM3. GLAC1D^43^ is 96 m higher at 11 ka compared to present and ICE-6G^44^ is 222 m higher. **b**, Temperature anomalies from elevation change (GLAC1D, solid lines; ICE-6G, dashed lines) using an atmospheric lapse rate of 9.8 °C km^−1^ and spatial lapse rates for interior West Antarctica of 12 °C km^−1^ (ref. ^45^) and 14 °C km^−1^ (ref. ^46^). **c**, HadCM3 residual-temperature anomalies for December (summer) calculated by subtracting the ORBIT run from the GLAC1D and ICE-6G runs in Fig. 2a, highlighting the portion attributable to changing elevation rather than insolation. **d**, Residual-temperature change in **b** subtracted from the results in **c**, showing the component driven from processes besides the direct lapse-rate (LR) effect and orbital forcing. Source data

Next, as boundary conditions in the HadCM3 simulations, we prescribed variable greenhouse gas (GHG) concentrations and two different ice-sheet histories, GLAC1D and ICE-6G, which entail net surface lowerings of about 83 m and about 208 m, respectively, from 11 to 7 ka at the WDC site (Fig. 4a). These elevation scenarios substantially affect simulated temperatures (Fig. 2a,c). Much of the elevation-induced warming in these models, which occurs primarily in the early Holocene, can be attributed directly to the surface lapse-rate effect (Fig. 4b). However, comparison to the orbital-only runs (Fig. 4c) reveals a remaining temperature anomaly (Fig. 4d), attributable to GHGs, ice-sheet extent and nonlinear responses to simultaneously imposed forcings. Sea ice has only a small impact on the temperature at 80° S in summer (Methods—Sea ice; Extended Data Fig. 6).

Inconsistencies exist between the different ice-sheet scenarios (Fig. 4d) and the summer versus winter seasons but differences are minor enough to permit a bounded estimate of the true Holocene elevation decrease. This calculation is made by comparing the excess of the reconstructed temperature increase over the orbital-only simulation to the same excess for the ice-sheet model simulations and scaling to the elevation changes used in the latter (Methods—Estimating elevation changes). We find central estimates for elevation decrease of 23 m and 53 m from comparison to the GLAC1D and ICE-6G scenarios, respectively, over the period 10 to 3.5 ka (Table 1). Accounting for uncertainties in the seasonal temperature reconstructions (Fig. 2) allows for elevation changes ranging from 33 m increase to 131 m decrease (2*σ*) from 10 to 3.5 ka or 54 m increase to 162 m decrease (2*σ*) if the time interval is narrowed to 10 to 6.5 ka (Table 1). Our results, thus, are consistent with geological observations of ice high-stands on mountain nunataks, which indicate less than 100 m of Holocene surface lowering^4–6^.

**Table 1:** WAIS elevation decrease

	GLAC1D			ICE-6G		
Interval	−2*σ*	Nominal	2*σ*	−2*σ*	Nominal	2*σ*
10–6.5 ka	−9.93	25.67	58.75	−54.00	57.52	161.96
10–3.5 ka	−5.63	22.99	48.49	−33.23	52.63	130.53

Elevation decrease estimates in metres (2*σ*, positive values correspond to a lowering ice sheet) for the intervals 10–6.5 ka and 10–3.5 ka (Methods—Estimating elevation changes).

Winter temperatures on the Antarctic mainland must respond to insolation forcings indirectly, via heat transport from lower latitudes. Orbital forcing models predict winter cooling across the Holocene, mostly from 11 to 6 ka (Figs. 2c and 3g). Both models and reconstructed winter temperatures lack a late Holocene maximum. But in the earlier Holocene, the winter reconstruction does not display the cooling trend expected from models and is dominated by prominent millennial variations. The mismatch with insolation at lower latitudes and absence of local forcings suggests variations in the efficacy of meridional atmospheric-heat transport.

## Discussion

Diverse and numerous proxies are used to reconstruct globally averaged surface temperatures for evaluating climate models and distinguishing natural from anthropogenic climate variability^33,34,36–38^. How these proxies depend on seasonal factors has been assessed in only a few cases^39^. Our West Antarctic study provides a cautionary example, as the mean annual temperature history reflects different controlling factors of summer and winter temperatures whose importance varies with time. In such a situation, important seasonal dynamics may be missed, or proxies misinterpreted, when only mean climate is considered. In addition, incorporating more information from the southern polar regions should help global temperature assessments avoid biases associated with weighting of temperature reconstructions toward northern sites, which have produced differing interpretations of the relationship between global climate and forcings in the Holocene, even including opposing trends^34,40,41^.

Previous analyses with simplified atmospheric models^3^ identified the duration of Southern Hemisphere summer as a key driving variable of Antarctic climate at orbital timescales. Some palaeoclimate findings validate this claim; for example, the onset of deglacial warming in West Antarctica corresponds with increasing integrated summer insolation^2^. Our results—spanning about half a precession cycle—reveal a dominant role for annual maximum insolation in determining West Antarctic summer climate during the Holocene, without precluding a greater role for duration or integrated summer insolation in other periods, such as glacial terminations.

## Methods

We measured WAIS Divide core (WDC) water isotopes using continuous-flow analysis (see next section on Water isotopes) and then corrected for cumulative diffusion using spectral techniques to determine diffusion lengths and restore prediffused amplitudes within 140-yr sliding windows (section below on Diffusion corrections; Extended Data Fig. 1c). Summer maxima and winter minima (Fig. 1a) identified in these corrected data were then used to calculate summer and winter amplitudes for each year. We converted the isotope amplitudes to temperature amplitudes using a model-determined scaling factor (section below on Seasonal temperatures) and added them to previously reconstructed mean annual temperatures^30^ to recover summer and winter values. Substantial seasonal noise processes required multicentennial to millennial averaging to reduce uncertainty (section below on Uncertainties in reconstructing temperatures). To elucidate physical controls on subannual temperatures, we used a simple energy balance model and HadCM3, a general circulation model, to calculate expected changes in seasonal and monthly surface temperatures through time under varying boundary conditions (sections below on Seasonal moist energy balance model and HadCM3 model simulations). Finally, using both observations and modelling, we estimated the change in WAIS surface elevation through the Holocene (section below on Estimating elevation changes).

### Water isotopes

WDC water isotopes (Extended Data Fig. 1a) were analysed on a continuous-flow analysis system^21^ using a Picarro cavity ring-down spectroscopy instrument, model L2130-*i*. Using permutation entropy^47^, we identified data anomalies arising from laboratory analysis, which were corrected, including by resampling ice through 1,035.4–1,368.2 m depths (4,517–6,451 yr)^48^. All other Holocene data are previously published^18,31^ and available online^19,20^. Data are reported in 5-mm increments in delta-notation (‰, or per mil) relative to Vienna standard mean ocean water (δ^18^O = δD = 0‰), normalized to standard light Antarctic precipitation (δ^18^O = −55.5‰, δD = −428.0‰). WDC is annually dated, with accuracy better than 0.5% of the age between 0 and 12 ka (ref. ^22^). For the Holocene, the temporal spacing of consecutive 5 mm samples is <0.1 yr and the average <0.05 yr, ranging from about 2.6 weeks at 10 ka to 0.5 week at 1 to 0 ka (ref. ^18^).

### Diffusion corrections

Diffusion in the firn and deeper ice attenuates high-frequency water-isotope information in ice cores^26,27,49–52^. Diffusion length quantifies the statistical vertical displacement of water molecules from their original position^27,49^. We used diffusion-correction code developed by S. Johnsen, University of Copenhagen^23,24,27,28^, which uses maximum entropy methods to invert an observed power-density spectrum. As an input to these inversions, we determined diffusion lengths (Extended Data Fig. 1c) for 140-yr windows using previous methods^18,26^. The power-density spectrum observed in the ice-core record $P\left(f\right)$,after diffusion, is $P\left(f\right)={P}_{o}\left(f\right){\rm{\exp }}\left[-{\left(2\pi f{\sigma }_{z}\right)}^{2}\right]$, in which ${P}_{o}\left(f\right)$ represents the power spectrum of the undiffused signal (‰^2^ m^−1^), $f$ is the frequency $\frac{1}{\lambda }$ (1/m), $\lambda$ the signal wavelength (m), *z* the depth (m) and ${\sigma }_{z}$ the diffusion length (m). The original, prediffusion power-density spectrum (diffusion-corrected) is calculated as ${P}_{o}\left(f\right)=P\left(f\right){\rm{\exp }}\left(4{\pi }^{2}{f}^{2}{\sigma }_{a}^{2}\right)$, for diffusion length ${\sigma }_{a}$ (yr) and $f$ now with units of 1/yr. The ${\sigma }_{a}=\frac{{\sigma }_{z}}{{\lambda }_{{\rm{avg}}}}$, in which ${\lambda }_{{\rm{avg}}}$ is the mean annual layer thickness (m yr^−1^) at a given depth. The diffusion-corrected spectrum takes the form of a series of complex numbers ${X}_{{\rm{R}}}+i{X}_{{\rm{I}}}$ versus $f$. From this, the amplitude spectrum $A$ is obtained by $A\left(f\right)=\sqrt{{X}_{R}^{2}+{X}_{{\rm{I}}}^{2}}$ and the phase spectrum $\varphi$ is obtained by $\varphi \left(f\right)={{\rm{\tan }}}^{-1}\left(\frac{{X}_{{\rm{I}}}}{{X}_{{\rm{R}}}}\right)$. The real components of the amplitude and phase spectrums give the diffusion-corrected water-isotope signal ${\delta }_{o}\left(t\right)$ as:

$${\delta }_{o}\left(t\right)=\mathop{\sum }\limits_{i=1}^{N}{A}_{i}{\rm{\cos }}\left(2\pi {f}_{i}t+{\varphi }_{i}\right)$$

Uncertainties on ${\delta }_{o}\left(t\right)$ are determined using the uncertainty range for diffusion lengths^26^ calculated in each 140-yr window. Before spectral analysis, the isotope data are linearly interpolated at a uniform time interval of 0.05 yr. Our determination of diffusive attenuation and correction arises from the observed frequency spectra themselves and therefore is entirely independent of firn diffusion and densification models.

#### Seasonal water-isotope amplitudes

To select extrema (summers and winters) in the diffusion-corrected δD signal (Fig. 1a and Extended Data Fig. 1b), we used the ‘findpeaks’ MATLAB function. Figure 1c,e show the resulting time series for summer and winter, averaged with a 50-yr boxcar filter for clarity of trends. For every year defined in the WDC age-scale, we calculated the averaged diffusion-corrected δD. The difference between the two extrema and the mean define the summer and winter isotope amplitudes.

#### Seasonal temperatures

A linear scaling converted seasonal isotopic amplitudes to seasonal temperature amplitudes, using a sensitivity of isotopes to surface temperatures determined by the simple water-isotope model (SWIM)^29^. Finally, to find summer and winter temperatures we added the individual seasonal temperature amplitudes to the year’s mean temperature obtained previously^30^ by calibrating the water-isotope record against borehole temperatures and δ^15^N constraints on firn thickness.

SWIM is based on earlier numerical Rayleigh-type distillation models^53,54^, which simulate the transport and distillation of moisture down climatological temperature gradients. As moist air is transported towards the poles and cools, the saturated vapour pressure decreases nonlinearly and moisture above saturation is removed by precipitation. The model keeps track of the isotopic fractionations at each step along this distillation process. In most previous simple models, there is an inconsistency in the calculation of the supersaturation that determines the point of condensation and that drives kinetic isotope fractionation. Modifications to these earlier models, used in SWIM, ensure consistency in the calculation, which results in a smoother relationship between temperature and the δ-values of precipitation and better agreement with observed spatial patterns of δD and δ^18^O. Given input of both δD and δ^18^O data, SWIM calculates distributions of source temperatures, the temperature gradients of pseudo-adiabatic pathways and condensation temperature. We used SWIM to derive sensitivities for surface isotope-temperature scalings using diffusion-corrected WDC data to obtain a surface scaling of 6.96‰ δD °C^−1^. Using raw data, the surface scaling is 7.07‰ δD °C^−1^. In comparison to other isotope-temperature scalings, ref. ^55^ obtain about 6.56‰ δD °C^−1^ and ref. ^30^ about 7.10‰ δD °C^−1^ (both converted from δ^18^O to δD using a factor of 8).

### Uncertainties in reconstructing temperatures

We included uncertainties associated with the following factors: measurement analysis, diffusion correction, seasonality of accumulation, precipitation intermittency, modelled isotope-temperature scaling and mean-temperature history. The ‘analysis uncertainty’ is 0.55‰ for δD (1*σ*) (ref. ^21^). The ‘diffusion-correction uncertainty’ is described in ref. ^26^. The uncertainty of the mean-temperature reconstruction, calculated previously^30^, accounts for most uncertainty in the early Holocene but a small fraction in the late Holocene. Sections ‘Seasonal preservation bias uncertainty’ to ‘Isotope-temperature scaling and associated uncertainty’ below explain the other uncertainty terms. Uncertainties for some factors (analysis and diffusion correction) can be treated as independent random variables so that, on time-averaging, their magnitudes decrease as the inverse of the square root of the number of values. Uncertainties for other factors (intermittency, isotope-temperature scaling, mean temperature and seasonality) might be systematically biased and therefore their magnitudes are taken to be invariant with respect to the interval of averaging. On the basis of the 2*σ* uncertainties for summer and winter temperature (Fig. 2a,c), we assessed the significance of dominant trends using Monte Carlo analysis (Fig. 2b,d; section on Trend analysis below).

#### Seasonal preservation bias uncertainty

**Extended Data Fig. 2:**
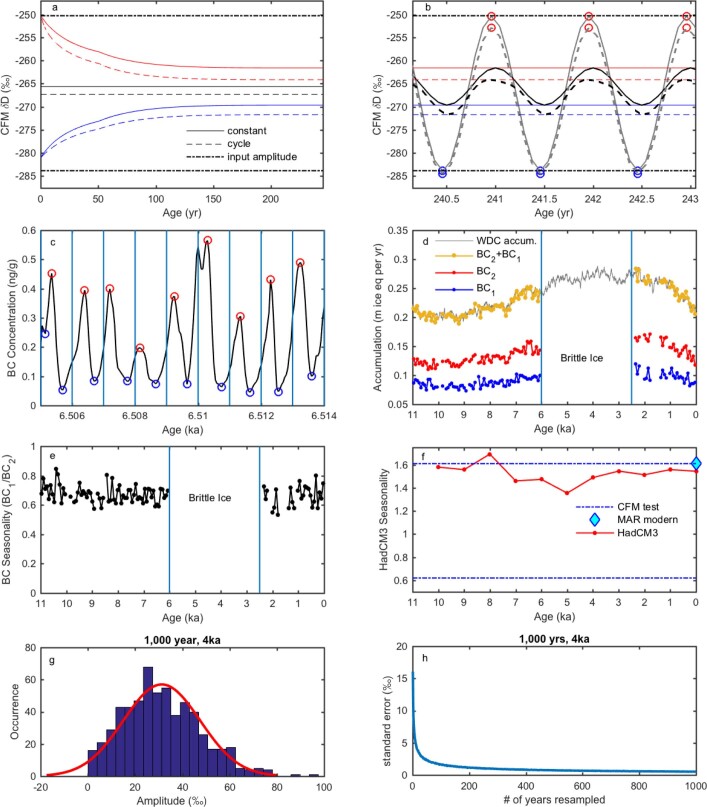
Precipitation uncertainties. Uncertainties for seasonally weighted accumulation are shown in a-f, and for precipitation intermittency in g-h. **a**, The diffusion envelope of CFM output data (50-yr avg.), based on an input sine wave with *f* = 1yr^−1^ and amplitude = 15.43‰. The original (input) amplitude of the signal (black, dotted-dashed lines) decreases as time passes due to diffusion and downward advection of the firn, as shown by the decay of the maximum (red) and minimum (blue) lines, while the mean values of the ‘constant’ and ‘cycle’ (black solid and dashed lines, respectively) scenarios do not change and are dependent on the seasonal weighting of snowfall. **b**, Diffused CFM output data from beneath the pore close-off depth (>200 yr) (black lines; smaller amplitude), with diffusion-corrected data shown with grey lines (larger amplitude). Red circles are the annual maximum value, and blue circles the annual minimum, selected using the same algorithm as Fig. 1a. **c**, A zoom of black carbon (BC) concentrations at ~6.5 ka^22,59^. The maxima (red circles) and minima (blue circles) can be used to separate approximate depth intervals corresponding to winter (BC_1_) and summer (BC_2_); vertical blue lines correspond to nominal January 1, as defined by the peak of nssS/Na^22^. **d**, The 140-yr averages for BC_1_ (blue) and BC_2_ (red). The grey line is WDC annual accumulation^76^, orange circles are BC_1_ + BC_2_, which should equal annual accumulation. **e**, Black carbon seasonality BC_1_/BC_2_ (black), based on (d). **f**, Accumulation seasonality for HadCM3 seasonal snowfall (red line) compared to the range of seasonality tested using the CFM (dashed blue lines) and modern MAR seasonality^58^ (blue diamond). **g**, Distribution of annual amplitudes for water isotopes for a 1,000-year window centred at 4 ka. **h**, Standard errors are determined for 1,000-realizations of random sampling of the distribution in (g) to determine a standard deviation of the residuals of the true mean minus the mean of the random sampling.

Unequal seasonal distribution of snowfall could result in different magnitudes of diffusion for winter and summer amplitudes^49^. The seasonal temperature cycle also affects the magnitude of diffusion for all seasons. We used the Community Firn Model (CFM)^56,57^, a firn-evolution model with coupled firn temperature, firn densification and water-isotope modules, to test how seasonally weighted accumulation affects the diffusion of specified, hypothetical isotope records progressing from surface snow (δD_snow_), to consolidated snowpack in the firn (δD_firn_), to solid ice beneath the pore close-off depth (δD_ice_). We applied the back-diffusion calculation (section on Diffusion corrections) to δD_ice_ to estimate the original δD_snow_. We then assessed how reconstructions of δD_snow_ could be misinterpreted as a result of different seasonal-accumulation weightings (Extended Data Fig. 2a,b).

We performed five CFM runs using monthly time steps for accumulation, temperature and isotopes (Extended Data Table 1). The seasonal cycle for δD_snow_ is based on the mean amplitude in Fig. 1b (15.43‰). Five WAIS accumulation scenarios were tested on the basis of monthly accumulation from the regional climate model MAR3.6 (Modèle Atmosphèrique Règional; ERA-Interim forced)^58^, which spans the period January 1979to December 2017. The mean accumulation over the entire 39-yr period is 0.24745 m ice equivalent yr^−1^, with about 1.6× as much snow in winter (April to September) as in summer (October to March). The five scenarios are as follows: (1) ‘constant’: identical accumulation for all months (0.0206 m ice equivalent month^−1^; one-twelfth of the annual mean); (2) ‘cycle’: monthly accumulation equal to MAR monthly means; (3) ‘noise’: using the ‘cycle’ time series, we add noise to each time step in the ‘cycle’ series in the form of a normal random variable of zero mean and the standard deviation for the month from MAR; (4) ‘random’: for each month, the accumulation is a normal random variable with mean and standard deviation equal to MAR monthly values; and (5) ‘loop’: the entire 39-yr MAR accumulation time series is repeated over and over again. For the temperature boundary condition, we used the mean temperature at a height of 2 m for 1979–2017 for each month predicted by MAR to create an annual temperature cycle. We repeated this 12-month time series for the duration of the model runs. This method ensures that model runs, which are designed to test accumulation seasonality, are not affected by interannual temperature variability, while also providing an estimate of the annual temperature cycle, which affects the rate of isotope diffusion in the upper firn.

Extended Data Fig. 2a,b shows the results for the ‘constant’ and ‘cycle’ cases. The diffusion-correction technique accurately reconstructs δD_snow_ for summer and winter in the ‘constant’ snowfall scenario but underestimates summer values in the MAR ‘cycle’ scenario by about 2.6‰, which is 11% of the full range of the observed WDC summer water-isotope values. Winter values are overestimated by only about 0.6‰, about 3% of the full winter range, since winter has 1.6× as much snow as summer. The ‘noise’, ‘random’ and ‘loop’ runs produce results within 0.3‰. These CFM experiments demonstrate that centennial trends in the summer and winter water isotopes of the order of a few per mil (‰) could arise from large changes in seasonal-accumulation weighting, whereas multimillennial trends ≫2.6‰ are unlikely to be caused by seasonal accumulation and can therefore be interpreted as climate signals of a different origin. For 1,000-yr averaging (as in Fig. 2), HadCM3 indicates seasonal-accumulation weighting (winter:summer) of 1.3 to 1.7 throughout the Holocene (Extended Data Fig. 2f), which yields a 1*σ* uncertainty of 0.27‰ based on the CFM testing criteria.

To determine observationally if seasonal snowfall changed across the Holocene, we used measured black carbon (BC) concentrations, the only age-scale-independent impurity. BC data are available from 0–2.5 ka and 6–11 ka (ref. ^22^). Seasonal-fire regimes in South America dominate BC concentrations at WDC, causing BC maxima and minima in autumn and spring, respectively^59^ (Extended Data Fig. 2c). We split each year into two parts, characterized by rising or falling BC: BC_1_ and BC_2_ the depth intervals of rising and falling BC (Extended Data Fig. 2d). The duration of BC_2_ is longer than BC_1_ owing to source characteristics^59^, thus BC_1_/BC_2_ < 1 (Extended Data Fig. 2e). The BC_1_/BC_2_ ratio can change with time because of variability at the source, changes in atmospheric transport or seasonality of snow deposition. We observe little change in BC_1_/BC_2_ resembling the multimillennial trends seen in WDC summers and winters (Extended Data Fig. 2e). Unless there are competing and exactly compensating effects in seasonality (the source change exactly cancels the depositional and transport change or other unlikely scenarios), the BC data provide evidence that changes in WDC seasonal snowfall were not large enough to affect our multimillennial climate interpretations.

#### Intermittency of precipitation uncertainty

The episodic nature of snowfall creates an incomplete record of local climate variations^60^, preventing interpretation of trends over short time intervals. We want to interpret isotopic variations averaged over a sufficiently long timescale so that, to within a specified tolerance, trends are not likely to be random noise arising from the spread of distributions preserved in the ice. Using distributions of reconstructed annual amplitudes (Fig. 1b and Extended Data Fig. 2g) for 1,000-yr windows throughout the Holocene, we conducted Monte Carlo resampling simulations to determine that 250-yr averaging-lengths are needed to achieve a standard error of 1‰, corresponding to a mean amplitude-to-noise ratio of 15. For the time period with greatest variability, centred on 4 ka, the standard error for a 1,000-yr average (as used in Fig. 2) is 0.52‰ (Extended Data Fig. 2h). Because this is an amplitude uncertainty (rather than uncertainty associated with a season), we specify the 1*σ* uncertainties for summer and winter as half of 0.52‰.

#### Isotope-temperature scaling and associated uncertainty

The conversion of isotopic values (1,000-yr averages) to temperature yields three curves for summer and three for winter: *T*_nominal_, *T*_+1*σ*_ and *T*_−1*σ*_. Each curve is normalized to the value at 1 ka (as done in Fig. 2). The difference in the *T*_+1*σ*_ and *T*_−1*σ*_ curves gives the 1*σ* uncertainty range +*σ*_Tscale_ to −*σ*_Tscale_, which are then added in quadrature to the ref. ^30^ mean-temperature uncertainties, yielding the final uncertainty estimates shown in Fig. 2.

#### Relationship between the annual mean and individual seasons

**Extended Data Fig. 3:**
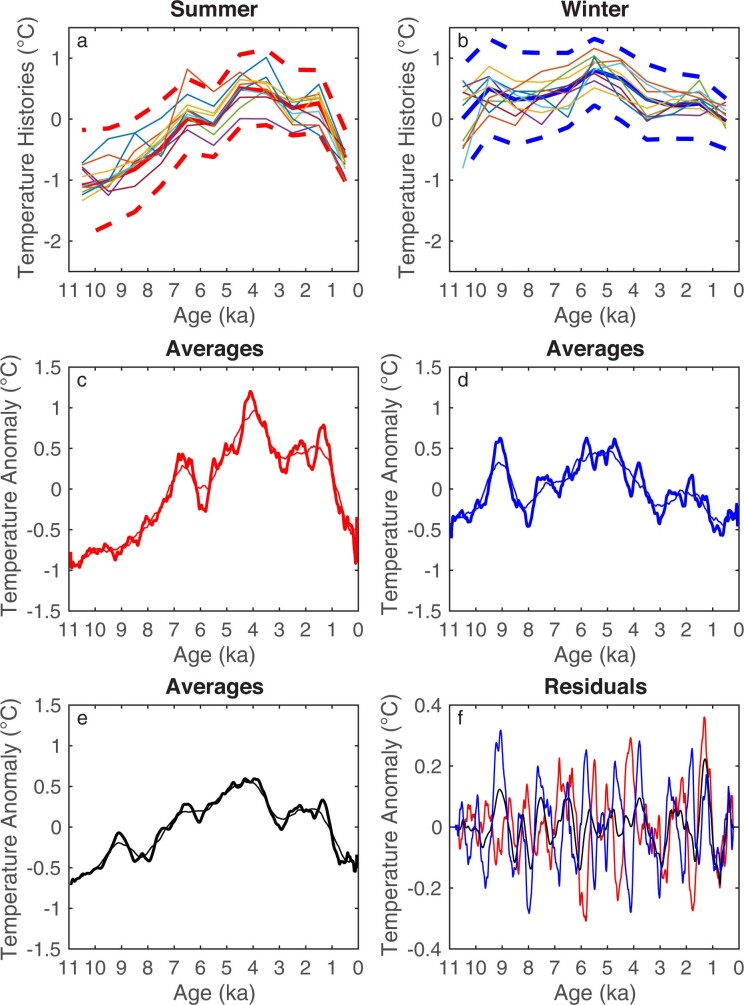
Trend analysis. **a**,**b**, The first 10 of 10,000 randomly generated, alternative seasonal temperature histories for summer (a) and winter (b), used in Fig. 2b,d to generate probability distributions of temperature trends in the Holocene. Thick, solid lines are mean values, and thick, dashed lines are 2*σ* uncertainty ranges. **c**–**f**, The 1,000-year (thin line) and 300-year (thick line) averages normalized to the 11-0 ka mean (c–e) and residuals (1,000 year minus 300 year) (f) of summer (red), winter (blue), and the mean (black), used to calculate R^2^ values in Extended Data Table 2.

Using 1,000-yr and 300-yr averages of summer, winter and mean temperature (Extended Data Fig. 3c–f), we determined *R*^2^ values for summer and winter versus the mean. We then subtracted the 1,000-yr averages from the 300-yr averages to obtain residuals and then determined *R*^2^ values again for summer and winter versus the mean (Extended Data Table 2). From 11 to 0 ka, the high summer correlations for the 1,000-yr comparison indicate a strong association of the annual mean temperature with the summer temperature at orbital timescales. At suborbital scales (300–1,000-yr residuals), neither the summer nor the winter alone explain much of the mean annual variability and the annual mean is a random composite of the two seasons. If only 11–7 ka is considered, winter variability explains more of the mean at submillennial scales.

#### Trend analysis

To assess the significance of dominant trends in our reconstructed seasonal temperatures, we conducted a Monte Carlo analysis founded on the assumption that all possibilities for the unknown time-dependence of errors are equally likely. The essentialmotivation for this approach is that we have determined the magnitudes of uncertainties as a function of age but that we have no information about whether the errors in our reconstruction persist at similar values for long periods of time (exhibit a bias) or whether they fluctuate at high-frequency.

We randomly generated a large number of alternative seasonal temperature histories governed by the uncertainties on 1,000-yr averages (Extended Data Fig. 3a,b), calculated the temperature trends for each alternative history over desired time intervals (such as 11–4 ka and 4–0 ka) and compiled the results into frequency distributions from which probabilities can be calculated (Fig. 2b,d). Specifically, each alternative history deviates from the summer and winter temperature reconstruction by an amount that smoothly varies over time between random nodes whose values are a Gaussian random variable of zero mean and standard deviation for 1,000-yr averages at the age of the node. The number of nodes and the age of each node are random variables, uniformly distributed between 1 and 11 nodes and 0–11 ka, respectively. A small number of nodes produce an alternative temperature history for which the bias is serially correlated for millennia, whereas a large number of nodes produce a history for which the bias is uncorrelated from millennium to millennium.

### Seasonal moist energy balance model

**Extended Data Fig. 4:**
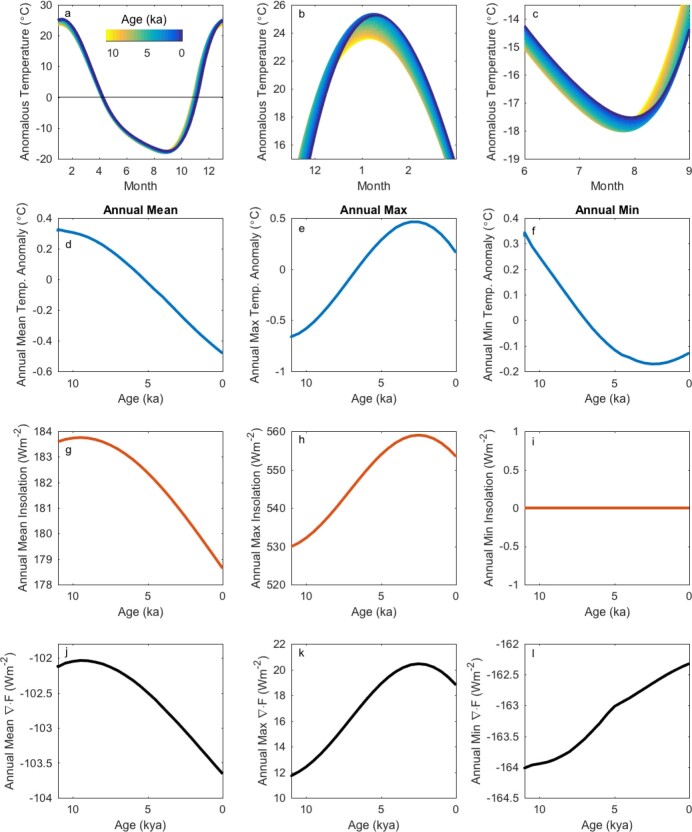
MEBM results. MEBM seasonal surface temperatures are shown in a–c. **a**, Modelled seasonal surface temperature cycle at 80°S, coloured by age. **b**,**c**, Zoom in of modelled summer and winter temperature. MEBM annual results for the mean, maximum, and minimum are shown in d–l. Plots for temperature anomaly normalized to the mean (blue), insolation (red), and heat divergence (black) for the annual mean (d,g,j), annual max (e,h,k), and annual min (f,i,l). Note the sign of heat divergence; negative values correspond to heat convergence at the site.

We used a simple global, zonal mean MEBM to calculate surface temperatures (Extended Data Fig. 4), accounting for TOA insolation, temperature-dependent longwave emission to space, temperature-dependent albedo to simulate brightening by snow and ice and horizontal atmospheric-heat transport treated as diffusion of near-surface moist static energy^61–63^. The model has a 2° spatial resolution, a single surface and single atmospheric layer and a surface-heat capacity based on the relative fraction of land and ocean surface in the zonal mean. Heat exchange between the surface and atmosphere layers arises from differences in blackbody radiation from each layer and sensible and latent heat exchanges proportional to temperature and specific humidity contrasts (assuming a constant relative humidity of 80%), following bulk aerodynamic formulae. We calculated the annual TOA insolation-cycle at every latitude in 500-yr time slices from 0 to 11 ka. For each time slice the model is run at 2 hour time resolution for 30 model-years to reach equilibrium.

Extended Data Fig. 4 compares the temporal evolution of summer maximum and winter minimum heat divergence by the atmosphere (∇·**F**) at the WDC site to the summer maximum and winter minimum site temperature and insolation. Although Holocene changes in ∇·**F** at the WDC site correlate with insolation forcing, the magnitude of changes in maximum direct insolation are much larger than those in atmospheric-heat divergence. Further, the Holocene changes in summertime heat divergence are of the wrong sign to cause net heating at the WDC site (positive divergence is an export of heat by the atmosphere from the site). Heat transport in the Antarctic is convergent in the annual mean but divergent in mid-summer, as intense incoming insolation exceeds longwave emissions from the cold surface.

### HadCM3 model simulations

#### Model setup

We used the fully coupled ocean-atmosphere model HadCM3 (refs. ^64,65^), v.HadCM3BM2.1, which well simulates tropical Pacific climate and its response to glacial forcing^66^. Our simulations are snapshots at 1 kyr intervals over the last 11 ka (ref. ^35^), with time-specific boundary conditions of orbital forcing^67^, GHG concentration^68,69^, ice-sheet topography and sea level^43,44,70–74^. We used three simulations: (1) only orbital forcing changes (ORBIT), with all other boundary conditions set to the pre-industrial; (2) orbital/GHG forcing with GLAC1D ice-sheet elevation history; and (3) orbital/GHG forcing with ICE-6G ice-sheet elevation history. Elevation histories are shown in Fig. 4a. Snapshot simulations were run for at least 500 yr with analysis made on the final 100 yr. Further snapshot simulations for 10 ka allowed us to decompose the role of different forcings, described in the following sections. The large difference in forcings between 10 ka and the pre-industrial late Holocene epoch make this comparison most instructive.

#### Summer climate

We examined the zonal mean at 80° S in simulations for hypothetical 10 ka worlds, by changing the boundary conditions to compare to the pre-industrial/late Holocene. These simulations are ‘10 ka ORBIT-only’; two runs with only ice sheets at 10 ka and pre-industrial settings otherwise, called ’10 ka GLAC1D-only’ and ‘10 ka ICE-6G-only’; and two runs with all 10 ka forcings, called ‘10 ka GLAC1D-all’ and ‘10 ka ICE-6G-all’. In ‘10 ka ORBIT-only’, reduced TOA shortwave radiation causes a large reduction in shortwave radiation at the surface (SW_d_) and consequent cooling. Downward longwave radiation (LW_d_) also decreases, probably because of atmospheric cooling. Sensible heat flux (SH_d_) to the surface is increased, indicating that the atmosphere and surface do not equally cool; one cause of this is the increased meridional heat convergence (−∇·**F**).

Changed ice sheets cause summertime cooling in both ‘10 ka GLAC1D-only’ and ‘10 ka ICE-6G-only’, primarily via reduced LW_d_. Increased SW_d_, as a result of reduction of depth of the atmospheric column above the ice-sheet surface, counteracts the reduced LW_d_ to some extent. (Reducing the atmospheric column reduces SW absorption and tends to cool the atmosphere, reducing LW_d_). Both ice-sheet scenarios also cause an increase in −∇·**F**, partly counteracting summertime cooling.

Using all 10 ka forcings causes cooling through both LW_d_ and SW_d_. The decrease in SW_d_ is similar in ‘10 ka GLAC1D-all’ and ‘10 ka ICE-6G-all’ and slightly smaller than in ‘10 ka ORBIT-only’, probably because the thinner atmospheric column reduces absorption. The decrease in LW_d_ in ‘10 ka GLAC1D-all’ and ‘10 ka ICE-6G-all’ is larger than in ‘10 ka ORBIT-only’, ‘10 ka GLAC1D-only’ and ‘10 ka ICE-6G-only’. This indicates the importance of feedbacks in the atmosphere. Heat convergence −∇·**F** increases in both simulations indicating remote feedbacks, in addition to local feedbacks related to the amount of water vapour in the atmosphere.

The preceding description of changes in the zonal mean in the 10 ka simulations compared to pre-industrial period holds for the entire Holocene epoch. Orbital forcing alone reduces SW_d_ and LW_d_ by roughly the same magnitude. With full forcing (including ice sheets), the reduction in LW_d_ is roughly three times the reduction in SW_d_. Considering an energy budget over the WDC site (79.467° S, 112.085° W), mechanisms are the same as for the zonal mean. Magnitudes of forcings change but reduced SW_d_ still cools the surface, amplified by an LW_d_ feedback dependent on ice-sheet size.

#### Winter climate

During winter, SW_d_ is no longer a factor as the sun is below the horizon, yet there is still surface warming caused by an increase in LW_d_. An increase in −∇·**F** in ‘10 ka ORBIT-only’ warms the atmospheric temperature, increasing LW_d_ and SH_d_. With an ice sheet imposed, the surface temperature cools. In both ‘10 ka GLAC1D-only’ and ‘10 ka ICE-6G-only’ there is a reduction in −∇·**F**, reducing LW_d_ and SH_d_. When all 10 ka forcings are introduced, the change in temperature is smaller than for ice sheet-only runs. In ‘10 ka GLAC1D-all’ we found no change in −∇·**F**, LW_d_ or surface temperature. This suggests that the increase in −∇·**F** from orbital forcing is almost perfectly balanced by the change in −∇·**F** from the ice-sheet configuration.

**Extended Data Fig. 5:**
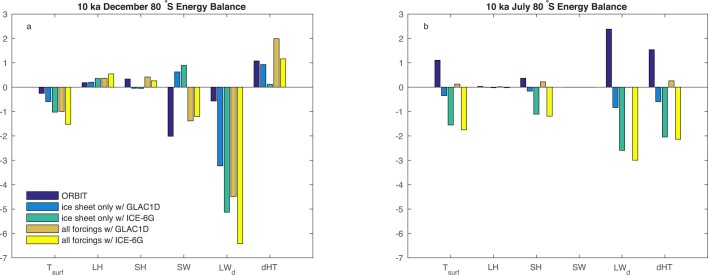
80°S energy balance at 10 ka. Bar charts of HadCM3 energy balance terms at 10 ka for **a**, December (summer) and **b**, July (winter), including model runs for ‘orbit only’ (purple, ORBIT), ‘ice sheet only’ (blue, GLAC1D; green, ICE-6G), and ‘all forcings’ (orange, GLAC1D; yellow, ICE-6G). Positive values all indicate a surface or atmospheric warming. Variables include surface temperature (T_surf_ in **°**K), latent heat (LH in Wm^−2^), sensible heat to the surface (SH_d_ in Wm^−2^), shortwave radiation (SW in Wm^−2^), downward LW radiation (LW_d_ in Wm^−2^), and change in heat transport (∇·**F** in 10^7^ W).

The processes controlling heat transport over Antarctica are complicated and HadCM3 may not be able to simulate them perfectly. Our simulations indicated that remote processes during winter alter the heat transport, affecting atmospheric and surface temperatures. Raising the topography of Antarctica tends to reduce such heat transport (Extended Data Fig. 5), producing an additional cooling on top of a pure lapse-rate effect^75^. This cannot, however, explain the prominent millennial-scale changes at about 9.2 and about 7.9 ka (Figs. 1e and 2c). The intricacies of interpreting the early Holocene winter variability in West Antarctica necessitates further study.

#### Sea ice

**Extended Data Fig. 6:**
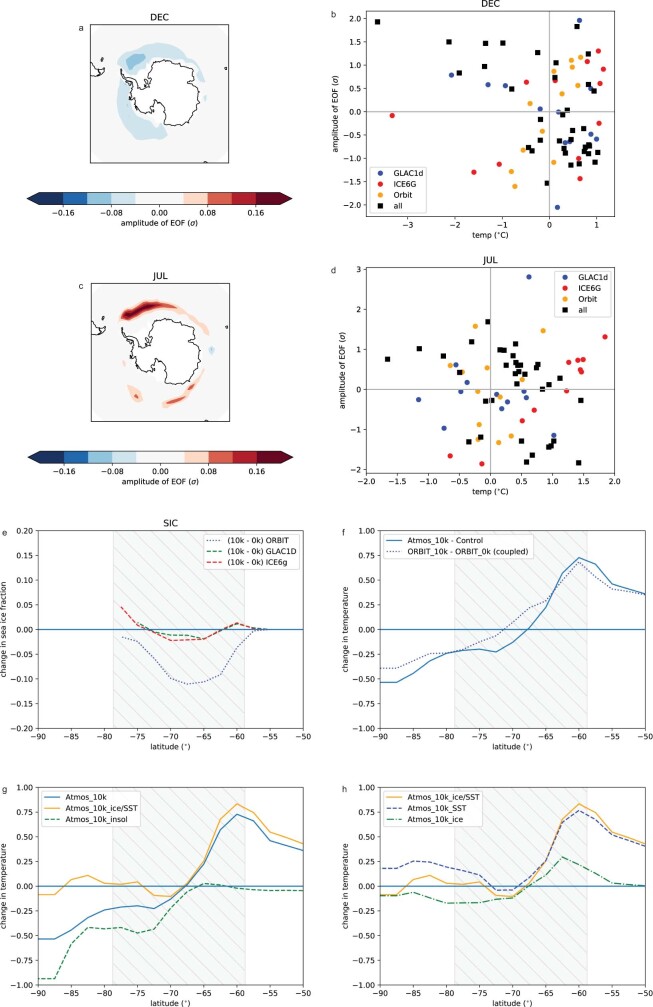
Sea ice variability and temperature in HadCM3 simulations. Maps of the dominant pattern of variability in each of the seasons and scatter plots of amplitude of the pattern against temperature. Maps were created using Python’s package cartopy. **a**,**c**, The EOF of sea ice variability in the Southern Hemisphere for December and July in ALL of the simulations: this is the dominant pattern of sea ice variability. **b**,**d**, The amplitude of the patterns in (a) and (c) vs. the temperature at 80°S for each simulation. Plots e–h show the zonal-mean temperature and sea ice in HadCM3 simulations for December average. **e**, Change in sea ice fraction for coupled-model simulations from 10 ka to 0 ka. **f**, Change in surface temperature between 10 ka and 0 ka for the coupled-model simulations (dotted line) and atmosphere-only simulations (solid line). **g**, Change in surface temperature for atmosphere-only runs from 10 ka to 0 ka. **h**, Change in surface temperature for atmosphere-only runs from 10 ka to 0 ka. See Extended Data Table 3 for descriptions of the model simulations used in panels (g) and (h).

**Extended Data Fig. 7:**
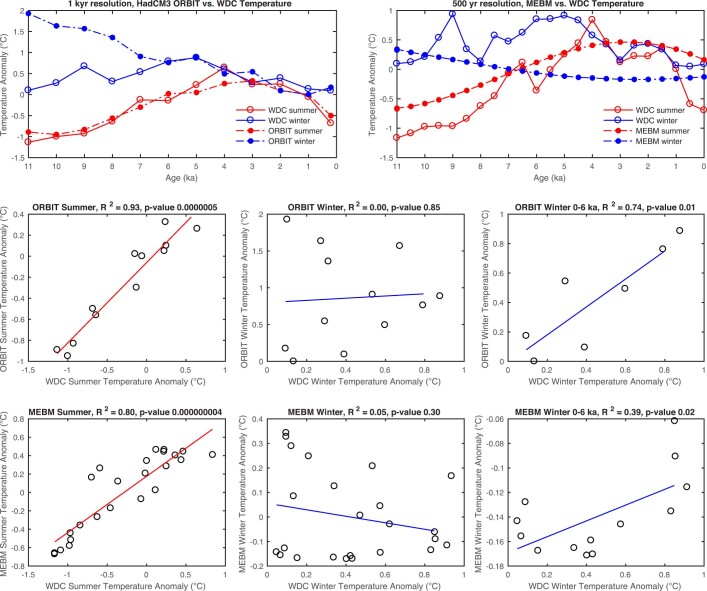
Model results vs. WDC temperatures. Coefficient of determination and p-values for comparison of HadCM3 (1-kyr resolution, n = 12) or MEBM (0.5-kyr resolution, n = 23) model results with WDC summer and winter temperatures.

Sea ice changes may alter local energy fluxes from the ocean to the atmosphere. In HadCM3, sea ice extent changes across the Holocene. We used two analyses (Extended Data Fig. 6) to show that sea ice is not a primary control on the surface temperature at WDC (80° S): (1) correlation analysis of sea ice changes and surface temperature and (2) atmosphere-only model simulations in which we specified individual changes in the model boundary conditions (including sea ice).

We computed the dominant spatial patterns of sea ice variability using empirical orthogonal functions across all of the HadCM3 simulations (ALL) and individually for three subset simulations (ORBIT, GLAC1D and ICE-6G), from 0 to 11 ka. We projected the model-simulated sea ice for each individual time-slice simulation onto these patterns to compute the amplitude of sea ice variability in each simulation. The amplitude was compared to temperature at 80° S to understand how large-scale changes in the sea ice affect temperature for the months of December (summer) and July (winter) (Extended Data Fig. 6).

In winter, we found negligible correlations between sea ice change and temperature in all sets of simulations (ALL: 0.02; ORBIT: 0.04; GLAC1D: 0.16; ICE-6G: 0.06). This suggests that winter sea ice is not an important factor in determining the temperature at 80° S. In summer, only the ORBIT simulation has meaningful correlations between temperature and sea ice variability (ALL: 0.59; ORBIT: 0.84; GLAC1D: −0.60; ICE-6G: 0.36). The sign of the correlation changes between simulations despite the sea ice change pattern being the same in all simulations. From this we concluded that sea ice is not a dominant control on temperature at 80° S in summer. The correlation in the ORBIT simulations suggests that there may be some relationship between sea ice and temperature; we investigated this with atmosphere-only simulations.

In atmosphere-only simulations at 0 ka and 10 ka, we specified the top of the atmosphere insolation, sea surface temperature (SST) and sea ice from the ORBIT simulations. Over land areas and sea ice regions, the model calculates the surface temperature using the land-surface scheme in the model. The atmosphere model is identical to the model used within the coupled model. We ran a series of experiments varying the orbital configuration, SST or sea ice (summarized in Extended Data Table 3).

The zonal mean of the change in the sea ice that we prescribed can be seen in Extended Data Fig. 6e and the change in the SST can be inferred from Extended Data Fig. 6f–h. Extended Data Fig. 6f shows that the atmosphere-only model replicates the change in temperature of the coupled model. Extended Data Fig. 6g shows that the effect of the 10 ka orbital configuration (‘Atmos_10k_insol’) is to cool Antarctica considerably by about 0.5 °C. North of 65° S there is no change in the surface temperature, primarily a response to the imposed SST and sea ice, which are the same in the ‘Control’ and ‘Atmos_10k_insol’. Imposing the SST and sea ice from 10 ka (‘Atmos_10k_ice_SST’), we find very little change in the surface temperature over Antarctica but there are some large changes in the surface temperature north of 70° S. Extended Data Fig. 6h shows the result of imposing 10 ka SST or 10 ka sea ice. The 10 ka SST (‘Atmos_10k_SST’) tends to warm Antarctica, consistent with the large increases in SST north of 65° S. Changing sea ice (‘Atmos_10k_ice’) tends to cool Antarctica. Both effects are small, approximately 0.1 °C and of opposite sign. This explains the small net change in the surface-temperature change over Antarctica when SST and sea ice are changed simultaneously, as shown by ‘Atmos_10k_SST_ice’. It should be noted that in the coupled system a change in sea ice cannot be decoupled from a change in the SST, so not only is the effect of sea ice on the climate small, it is also probably associated with a compensating change in SST. From these simulations we concluded that sea ice and SST changes are not a dominant driver of the change in the surface temperature over Antarctica.

Extended Data Fig. 6e shows that the change in sea ice at 10 ka in ORBIT is much larger than the change in either GLAC1D or ICE-6G. The ORBIT simulations do not account for all of the changes in the boundary conditions at 10 ka and are therefore less realistic than either ICE-6G or GLAC1D. Because ICE-6G and GLAC1D both show much smaller changes in the sea ice and SST than ORBIT, we expect that in reality there is also a much smaller change in the sea ice and SST than in ORBIT. We thus concluded that sea ice has a small impact on the temperature at 80° S in summer.

We also performed a similar analysis of the winter season (not shown). We found that the atmosphere-only model does not compare well with the coupled model, simulating very little change in the surface temperature. Doing a term-by-term decomposition of the atmosphere model is not, therefore, particularly useful as it tells us more about the model rather than the physical climate. The failure of the atmosphere-only model to capture the changes at 10 ka suggests that the importance of the SST and sea ice is in their day-to-day coupling with the atmosphere and not in any long-term mean change in this season.

### Estimating elevation changes

Temperatures simulated by HadCM3 for ORBIT provide a control scenario against which observations can be compared to identify the signal of elevation change. For a chosen time interval, the net reconstructed warming Δ*T*_R_ exceeds that of ORBIT by an amount Δ*T*_R_ − Δ*T*_O_. This can be compared to the effective lapse rate (Δ*T*_M_ − Δ*T*_O_)/Δ*Z*_M_ defined by a HadCM3 simulation including topographic change (GLAC1D or ICE-6G models) and all forcings, for model warming Δ*T*_M_ and model elevation decrease Δ*Z*_M_. Specifically, the estimated elevation decrease is Δ*Z*_R_ = Δ*Z*_M_((Δ*T*_R_ − Δ*T*_O_)/(Δ*T*_M_ − Δ*T*_O_)). Summer and winter reconstructions offer two separate assessments, for which we calculate the algebraic average.

Accounting for uncertainties in Δ*T*_R_ requires recognizing that uncertainties of summer and winter reconstructions are not independent, while also recognizing that they emerge from two independent sources: uncertainty in the mean annual temperature history (calculated in ref. ^30^) and uncertainty in the seasonal amplitude (calculated in the present study). In general, the uncertainty of seasonal temperature at a specified time is the quadrature sum of annual and amplitude uncertainties, which gives 1*σ* and 2*σ* uncertainties for a given season and time. However, if the true value of annual temperature is shifted by an amount *ασ* from the nominal reconstruction, this must be true for both summer and winter. And if the true value of amplitude is shifted by an amount *βσ* from the nominal reconstruction, the temperature shift must be +*βσ* in one season but −*βσ* in the other.

To define bounding cases on elevation change in a specified time interval, we calculated the maximal (or minimal) temperature change Δ*T*_R_ for a season by differencing the upper (or lower) limit at one end of the interval with the lower (or upper) limit at the other end and also calculating the corresponding Δ*T*_R_ for the opposite season required by the correlated errors. The elevation decreases Δ*Z*_R_ were then calculated by comparison to HadCM3 simulations, as specified previously and the summer and winter values averaged. This process was completed four times for each time interval and HadCM3 model, corresponding to four different initial Δ*T*_R_ (maximum and minimum Δ*T*_R_ for summer and maximum and minimum Δ*T*_R_ for winter) and the most extreme case taken as the result (this proved to be the one starting with maximum summer Δ*T*_R_). Table 1 lists results for two time intervals and the two HadCM3 simulations with variable topography.

## Online content

Any methods, additional references, Nature Portfolio reporting summaries, source data, extended data, supplementary information, acknowledgements, peer review information; details of author contributions and competing interests; and statements of data and code availability are available at 10.1038/s41586-022-05411-8.

## Source data

Source Data Fig. 1

Source Data Fig. 2

Source Data Fig. 3

Source Data Fig. 4

Source Data Extended Data Fig. 1

## Figures and Tables

**Extended Data Table 1:**
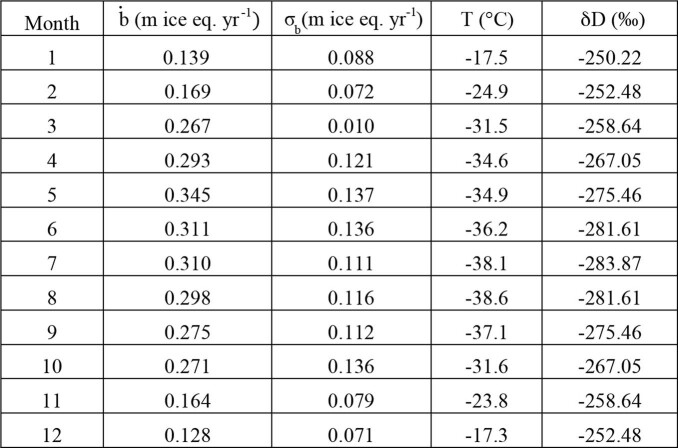
CFM simulation inputs

Mean $\mathop{{\rm{b}}}\limits^{.}$ and standard deviation ${{\sigma }}_{\dot{{\rm{b}}}}$ of each month’s accumulation rate and monthly mean temperature at WAIS Divide for 1979 to 2017 predicted by MAR^56^ and the isotope values used for each month during the CFM simulations.

**Extended Data Table 2:**
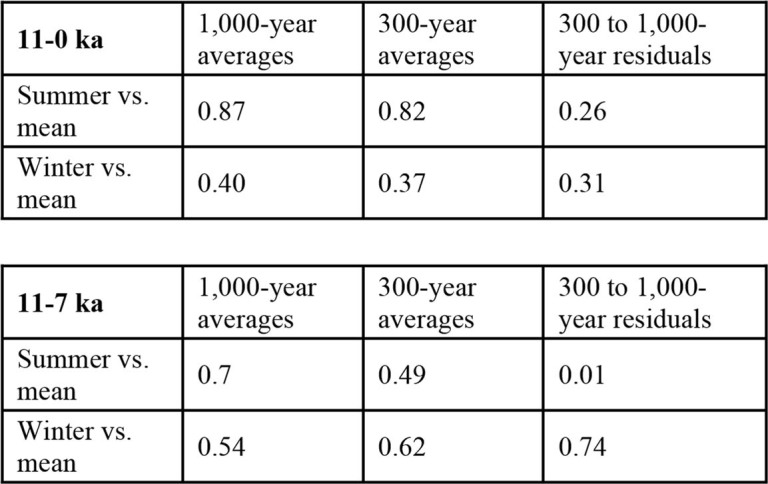
Seasonal vs. mean correlations

R^2^ values for 1,000-year and 300-year averages of summer and winter vs. mean, as well as for residuals.

**Extended Data Table 3:**
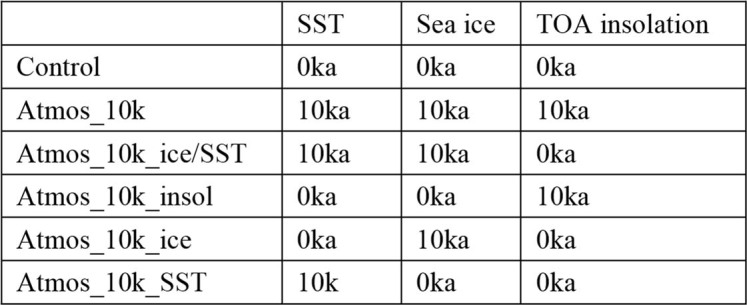
Atmosphere-only climate model experiments

Variations of boundary conditions in each experiment.

## Data Availability

The WDC water-isotope datasets analysed during the current study are available in the US Antarctic Program Data Center (USAP-DC) repository, 10.15784/601274 and 10.15784/601326. The impurity datasets analysed during the current study are available in USAP-DC repository, 10.15784/601008. The data generated in this study are available in the USAP-DC repository, 10.15784/601603, including raw and diffusion-corrected water isotopes, seasonal water isotopes (maximum summer and minimum winter values) and seasonal temperature reconstructions. Source data are provided with this paper.

## Code availability

The MATLAB code used for the diffusion correction of water-isotope data and the subsequent selection of seasonal extrema (summer and winter) are available online at Zenodo, 10.5281/zenodo.7042035.
